# Breast Cancer Cell Subtypes Display Different Metabolic Phenotypes That Correlate with Their Clinical Classification

**DOI:** 10.3390/biology10121267

**Published:** 2021-12-03

**Authors:** Consuelo Ripoll, Mar Roldan, Maria J. Ruedas-Rama, Angel Orte, Miguel Martin

**Affiliations:** 1 Nanoscopy-UGR Laboratory, Departamento de Fisicoquimica, Unidad de Excelencia de Química Aplicada a Biomedicina y Medioambiente, Facultad de Farmacia, Universidad de Granada, Campus Cartuja, 18071 Granada, Spain; consueloripoll@ugr.es (C.R.); mjruedas@ugr.es (M.J.R.-R.); 2 GENYO, Pfizer-Universidad de Granada-Junta de Andalucia Centre for Genomics and Oncological Research, Avda Ilustracion 114, PTS, 18016 Granada, Spain; mdmar.roldan@gmail.com; 3 Departamento de Bioquímica y Biología Molecular I, Facultad de Ciencias, Universidad de Granada, Avda. Fuentenueva, 18071 Granada, Spain

**Keywords:** metabolic profiling, breast cancer, tumor metabolism, metabolic reprogramming

## Abstract

**Simple Summary:**

Recent studies on cancer cell metabolism have achieved notable breakthroughs that have led to a new scientific paradigm. How cancer cell metabolic reprogramming is orchestrated and the decisive role of this reprogramming in the oncogenic process and tumor adaptative evolution has been characterized at the molecular level. Despite this knowledge, it is essential to understand how cancer cells can metabolically respond as a living whole to ensure their survival and adaptation potential. In this work, we investigated whether different cancers and different subtypes display different metabolic phenotypes with a focus on breast cancer cell models representative of each clinical subtype. The potential results might have significant translational implications for diagnostic, prognostic and therapeutic applications.

**Abstract:**

Metabolic reprogramming of cancer cells represents an orchestrated network of evolving molecular and functional adaptations during oncogenic progression. In particular, how metabolic reprogramming is orchestrated in breast cancer and its decisive role in the oncogenic process and tumor evolving adaptations are well consolidated at the molecular level. Nevertheless, potential correlations between functional metabolic features and breast cancer clinical classification still represent issues that have not been fully studied to date. Accordingly, we aimed to investigate whether breast cancer cell models representative of each clinical subtype might display different metabolic phenotypes that correlate with current clinical classifications. In the present work, functional metabolic profiling was performed for breast cancer cell models representative of each clinical subtype based on the combination of enzyme inhibitors for key metabolic pathways, and isotope-labeled tracing dynamic analysis. The results indicated the main metabolic phenotypes, so-called ‘metabophenotypes’, in terms of their dependency on glycolytic metabolism or their reliance on mitochondrial oxidative metabolism. The results showed that breast cancer cell subtypes display different metabophenotypes. Importantly, these metabophenotypes are clearly correlated with the current clinical classifications.

## 1. Introduction

In recent years, our concepts and knowledge of cancer cell metabolism have experienced a profound breakthrough that has led to a new scientific paradigm [1,2,3]. Since 2007, ground-breaking works have conceptually and contextually advanced tumor metabolism, which is currently considered a cancer hallmark [4,5,6,7].

In this context, a foundational scientific proposition is the concept of tumor metabolic reprogramming. This proposition was based on crucial experimental observations, such as the apparently paradoxical catalytic inefficiency of the glycolytic enzyme pyruvate kinase M2 (PKM2), which is expressed only in cancer and proliferative cells, relative to the more efficient PKM1, which is expressed in nontransformed somatic cells. PKM2 catalyzes the last glycolysis reaction; therefore, glycolytic flux is modulated by its lower catalytic velocity, which acts as a limiting factor. Consequently, many glycolytic intermediaries accumulate and can be used in branching anabolic routes, thus guaranteeing the high demand of biomass to sustain cell proliferation [8,9,10,11]. Similarly, alternative pathways for glutamine anabolic metabolism have been shown [12,13,14], as well as the prominent activity of the oxidative pentose phosphate pathway to supply NADPH for biosynthetic and antioxidant purposes [15]. In addition, important contributions have described the mechanisms by which amino acid and nucleotide biosynthetic pathways can be reprogrammed by alternative and preferential routes according to the cancer cell type, oncogenic progression and environmental adaptations [12,16,17]. All of these experimental findings have been decisive to establishing this new paradigm, in which the metabolism of cancer cells is not an “accident” due to mitochondrial dysfunction that provokes a high rate of aerobic glycolysis (Warburg effect) to guarantee energy demand but rather a response to highly efficient and selective mechanisms of adaptation to satisfy their high anabolic demand and sustain cell proliferation [3,7,18].

Metabolic reprogramming represents an orchestrated network of evolving adaptations during oncogenic progression. Cancer cells can adopt different metabolic landscapes in response to their own oncogenic evolution, changes in the cellular niche and environment, and resistance responses to chemo- and radiotherapy [18,19]. Importantly, oncogenes, such as KRAS, PI3K, Akt, and c-MYC, and tumor suppressor genes, such as p53 and AMPK, have been found to be crucial players that orchestrate the network of metabolic adaptations [19,20,21,22,23]. In addition, metabolic evolving adaptations further interact with cell signaling and epigenetic pathways, which in turn influence cancer progression. In line with these findings, a study recently proposed that metabolic reprogramming varies not only between different types of cancer but also between different clinical subtypes of the same tissue origin [24,25,26]. In this context, interesting works have been recently published on the metabolic implications for breast cancer clinics. Among these pioneering works, the relevance of differential profiles of expression markers for key metabolic targets between breast cancer subtypes has been discovered [27,28,29,30]. In addition, the prognostic and diagnostic application of some metabolic markers has been studied in breast cancer samples [31,32,33,34] as well as for the development of metabolism-based therapeutic strategies in breast cancer models [35,36,37,38]. Nevertheless, nearly all potential correlations between functional metabolic features and their clinical classification from a systemic perspective have not yet been fully studied.

In line with this research demand, we recently developed a novel metabolic nanosensor for robust measurements of mitochondrial pH. Interestingly, we found significant variations in intramitochondrial pH among different breast cancer cell lines that correlated with the current clinical subtype classification. Moreover, the variations in pH also correlated with differential responses to some metabolic drugs [39]. In a second work, we observed different sensitivity responses of breast cancer models to a chimeric small molecule developed for delivering the pyruvate dehydrogenase kinase inhibitor dichloroacetate to mitochondria [40].

Nevertheless, there are still relevant open questions from a functional perspective of breast cancer metabolic reprogramming. In this context, it is particularly interesting to investigate whether distinct metabolic profiles could correlate well with breast cancer clinical subtypes. The potential results from this type of investigation might have significant translational implications at different levels. Accordingly, the correlation between the current classification criteria for breast cancer subtypes and well-defined metabolic profiles could profoundly enhance the diagnostic and prognostic capacities. In addition, such work could pave the way for the development of novel therapeutic strategies based on “antimetabolic” subtype-specific drugs as well as novel technical applications with diagnostic and therapeutic purposes.

Recently, foundational contributions initiated the investigation of key metabolic features in different breast cancer cell models in vitro and in vivo. In this context, the characterization of glutamine dependence tested by differential sensitivity responses to transaminase inhibitor aminooxyacetate (AOA) was of particular relevance. Interestingly, these results were in correlation with the oncogene c-Myc expression levels between breast cancer cell lines representative of clinical subtypes [41]. In addition, the impact of AOA as a potential antimetabolic drug in breast cancer has been studied [42,43]. Likewise, opening works have shown some features of molecular impact and sensitivity responses to glutamate dehydrogenase (GDH) inhibitor epigallocatechin gallate (EGCG) [44,45], and the mitochondrial respiratory complex I inhibitor phenformin [46,47,48].

Moreover, there have been important contributions aiming to address whether breast cancer cell subtypes might feature different metabolic phenotypes. Most of these elegant works were performed by unlabeled metabolomic analysis to obtain quantitative data about total metabolite content. Some noticeable results were obtained but the data were not totally concordant displaying some discrepancies [49,50,51,52]. As indeed argued by some of the authors, unlabeled metabolome content analysis cannot address quantitative dynamic metabolic flux differences [49]. Accordingly, metabolome levels can locally and/or temporally change even in the absence of an alteration in metabolic fluxes. Unlabeled metabolome meta-analysis experiments with breast cancer cell lines, including hierarchical clustering and PLSDA, revealed interesting differences in glucose uptake between breast cancer subtypes. Moreover, it was also suggested possible differences in some central metabolic pathways such as amino acid metabolism and TCA [49]. Despite the valuable knowledge provided by these results, whether breast cancer cell subtypes might display different metabophenotypes in concordance with differences in central metabolic flux dynamics remained unresolved yet. For instance, it was shown how breast cancer cell subtypes can display a common phenotype of high glycolytic uptake and even similar dependence; however, how glucose could be preferentially diverted into one or another metabolic pathways has not been precisely addressed yet [49,51].

Under these premises, as already proposed by the aforementioned authors, further steps to address metabolic phenotypic differences between breast cancer cell subtypes need to be performed by dynamic metabolic flux analysis. In this sense, isotope labeling experiments are required to identify dynamic flux differences in central metabolic routes, such as glucose preferential usage and TCA reliance, as well as to decipher key enzymatic activities promoting those preferential pathways.

Notwithstanding the foundational and valuable information accounted by the aforementioned works, further steps are required in breast cancer cell metabolism research, particularly to accomplish the investigations about phenotypic and molecular metabolic differences among breast cancer cell subtypes.

Accordingly, the present work was designed to gain further insights in that direction. First, breast cancer cell lines were profiled by data combined from different treatments with five metabolic inhibitors: AOA, EGCG, phenformin, the PI3K inhibitor BKM120, and the mitochondrial membrane uncoupler BAM15. This profiling approach, testing the five selected inhibitors rather than profiles based on a single drug-single metabolic question design, was accomplished as a combinatorial profile aiming to evaluate systemic functional responses of breast cancer cell lines representative of each clinical subtype. Thus, following some insights provided by previous works, where single experimental treatments with AOA, EGCG or Phenformin were performed, in the present study the degree of glycolytic flux dependence was tested by sensitivity responses to phenformin and BKM120 treatments. In addition, the reliance on tricarboxylic acid cycle (TCA) activity and its connection with the potential preferences for cytosolic versus mitochondrial energy supply was studied combining data by AOA, BAM15 and EGCG treatments. Secondly, potential dynamic metabolic flux differences between breast cancer cell lines were studied by stable-isotope dynamic labeling for key targets, such as pyruvate, based on preferential pyruvate dehydrogenase (PDH) or pyruvate carboxylase (PC) activities to quantitatively analyze the degree of TCA reliance, and other central pathways as glutamine/glutamate metabolism preferences based on anaplerotic input to TCA or their preferential use by reductive carboxylation.

The results confirmed that there are differential dependences on glycolitiyc flux activity and TCA and anaplerotic activity reliance between hormone receptor-positive and triple-negative breast cancer subtypes. In addition, the cell lines representative of these clinical subtypes showed different dependencies on transaminase or glutamate dehydrogenase activity for recycling nonessential amino acids. Interestingly, the metabolic phenotypes were in correlation with the different breast cancer subtypes. Finally, as a mechanistic metabolic proof of principle, differences were found between hormone receptor-positive and triple-negative representative cell lines for preferential flux through pyruvate dehydrogenase (PDH) or pyruvate carboxylase (PC) by isotope labeling dynamic flux analysis. In addition, different preferences were also found in glutamine/glutamate paths between mitochondrial anaplerotic oxidative TCA recycling or cytosolic reductive carboxylation.

## 2. Materials and Methods

### 2.1. Cell Culture

Five breast cancer cell lines were selected, with each representative of a clinical subtype: MCF-7 (ER+PR+ve/HER2-negative); ZR751 (ER+PR+/HER2-negative); SKBR3 (ER/PR-negative/HER2+ve); MDA-MB-231 (ER/PR/HER2-negative or triple-negative); MDA-MB-468 (ER/PR/HER2-negative or triple-negative) [27,53,54]. MCF7 (HTB-22), MDA-MB-231 (CRM-HTB-26), and SKBR3 (HTB-30) cells were acquired from the American Type Culture Collection (ATCC, Manassas, VA, USA), and MDA-MB-468 (ACC 738) and ZR751 (ACC 8701601) cells were obtained from the Leib-Niz-Institut DMSZ German collection of microorganisms and cell cultures GmbH (DMSZ, Braunschweig, Germany).

The cell lines were subcultured every 72–96 h. Cell culture medium was refreshed the day before subculture. Cells were routinely plated in T75 flasks. The MCF7 cell line was cultured with minimum essential media (MEM, GIBCO) for optimal growth. MEM media was reconstituted with 10% fetal bovine serum (FBS, GIBCO), 1% GlutaMAX (GIBCO), 1% nonessential amino acids (NEAA, GIBCO) and 1% penicillin/streptomycin (P/S, GIBCO). Cells were routinely subcultured at a density of 1.8 × 10^4^ cells/cm^2^ for 72 h of culture or 1.46 × 10^4^ cells/cm^2^ for 96 h. The SKBR3 cell line was cultured with McCoy’s 5A medium for optimal growth (Sigma). Culture media was reconstituted with 10% FBS, 1% GlutaMAX and 1% P/S. SKBR3 cells were routinely subcultured at a density of 1.8 × 10^4^ cells/cm^2^ for 72 h of culture or 1.46 × 10^4^ cells/cm^2^ for 96 h. The MDA-MB-231 and MDA-MB-468 cell lines were cultured with Dulbecco’s modified Eagle’s medium with GlutaMAX (DMEM, GIBCO). DMEM was reconstituted with 10% FBS and 1% P/S. MDA-MD-231 cells were subcultured at a density of 1.8 × 10^4^ cells/cm^2^ for 72 h of culture or 1.46 × 10^4^ cells/cm^2^ for 96 h. MDA-MD-468 cells were subcultured at a density of 1.6 × 10^4^ cells/cm^2^ for 72 h of culture or 1.2 × 10^4^ cells/cm^2^ for 96 h. The ZR751 cell line was cultured with a slightly different protocol. This cell line has a longer doubling time; thus, ZR751 cells were regularly subcultured once a week, although the culture medium was changed twice between each cell seeding. ZR751 cells were cultured with RPMI 1640 (Sigma) for optimal growth. RPMI was reconstituted with 10% FBS, 1% GlutaMAX, 1% sodium pyruvate (NaPyr, GIBCO), and 1% P/S. The cells were habitually subcultured at a density of 2.4 × 10^4^ cells/cm^2^ in T75 flasks.

Each cell line was cultured strictly following the directions and protocol provided by the commercial brand suppliers to guarantee their optimal growth. The goal of this work was to study differential responses to metabolic inhibitors, and to perform dynamic flux analysis of metabolic activities and paths by isotope labeling approaches. Accordingly, the culture of all cell lines under the same media formulation have would affect their optimal proliferative and growth capacity, and thus possibly masking the results by an undesired challenging effect. Nonetheless, the original cell culture formulations provided by brand indications are supplied within optimal molar ranges for glucose, glutamine, pyruvate, amino acids, among other metabolites.

### 2.2. Metabolic Drugs

The transaminase inhibitor aminooxyacetate (AOA), glutamate dehydrogenase inhibitor epigallocatechin gallate (EGCG), mitochondrial complex I inhibitor phenformin hydrochloride (Phem), and mitochondrial membrane uncoupler BAM15 were obtained from Sigma-Aldrich. The PI3K inhibitor BKM-120 was obtained from Caymanchemic. All the drugs were prepared following brand-protocol instructions. Concentrated stock solutions were routinely aliquoted and stored at −20 °C. Working solutions were freshly prepared for each set of experiments.

### 2.3. Cell Treatments and Viability Assays

Experiments were performed in real time by directly seeding the cells in flat bottom black 96-well plates for further fluorescence analysis. Each cell line was seeded in the wells with their corresponding culture medium. Specifically, MCF7 cells were seeded at 1 × 10^4^ cells/well, whereas the cell lines ZR751, SKBR3, MDA-MB-231 and MDA-MB-468 were seeded at 8 × 10^3^ cells/well. All the cell lines were maintained in the wells with 100 μL of their corresponding culture medium. After plating, the metabolic drugs were added, and the plates were incubated for 96 h at 37 °C.

After 96 h of treatment, cell viability was monitored using a CellTiter-Blue^®^ cell viability assay (Promega) under the conditions described in each experiment. CellTiter was assessed according to the manufacturer’s protocol, and the fluorescence amount obtained was proportional to the number of viable cells. Viability was expressed with respect to the percentage of untreated cells (100%). Blank wells were included on each plate to measure the fluorescence from serum-supplemented culture medium in the absence of cells. Then, the cell plates were incubated for 20 min at 37 °C, and the fluorescence was measured in a GloMax^®^-Multi+ Detection System (Promega).

All experiments were performed five independent times in quadruplicate replicas for each experimental condition. Data analysis was performed by calculating the average value for all replicas of each experimental condition, and then media-blank values were subtracted from averages. Finally, data were expressed as percentages of viability relative to control untreated cells (100%).

### 2.4. Isotope Labeling and Metabolite Extraction

Cells were pulsed with heavy isotopes (Isotec, Sigma) dissolved in the corresponding routine media for MCF7 and MDA-MB-231 cell lines. Individual wells of a 6-well plate were washed with ice-cold PBS and then extracted in 0.5 mL extraction buffer (50% methanol, 30% acetonitrile, 20% water at −20 °C or lower). Extracts were centrifuged at maximum speed and stored at −80 °C.

### 2.5. LC-MS Analysis

LC-MS was carried out using a Thermo Ultimate 3000 HPLC in line with a Q Exactive mass spectrometer. A 32 min gradient was developed over a 100 mm × 4.6 mm ZIC pHILIC column with a guard column (Merck-Millipore) from 10% buffer A (20 mM ammonium carbonate) and 90% buffer B (acetonitrile) to 95% buffer A and 5% buffer B. Samples were acquired in positive-negative switching mode, and a standard ESI source and spectrometer settings were applied (typical scan range of 75–1050 Da). Metabolites were identified by standard metabolite matching to m/z and retention time. Integrated peak areas and label incorporation were quantified using AssayR [55].

## 3. Results

We have recently found that breast cancer cells representative of the clinical subtypes featuring positive expression to hormone receptors or triple-negative expression displayed significant differences in mitochondrial pH [39]. Mitochondrial pH values were obtained by a novel metabolic nanosensor developed and tested by our laboratory. Interestingly, our data pointed to a different metabolic pattern that opened the question of whether breast cancer cells could present different metabolic signatures. Accordingly, in the present work, we sought to address whether metabophenotypes would correlate with the current classification of the breast cancer clinical subtypes.

Changes in breast cancer cell metabolism have already been documented, and foundational works have already shown differential responses to single metabolic inhibitor treatments, metabolite availability selective conditions, as well as by metabolomic studies primarily using unlabeled total metabolite content analysis. Accordingly, following some insights provided by previous works, in the present study the degree of glycolytic flux dependence was tested by sensitivity responses to phenformin and BKM120 treatments [56,57]. In addition, the reliance on tricarboxylic acid cycle (TCA) activity and its connection with the potential preferences for cytosolic versus mitochondrial energy supply was studied combining data by AOA, BAM15 and EGCG treatments. Secondly, potential dynamic metabolic flux differences between breast cancer cell lines were studied by stable-isotope dynamic labeling for key targets, such as pyruvate, based on preferential pyruvate dehydrogenase (PDH) or pyruvate carboxylase (PC) activities to quantitatively analyze the degree of TCA reliance, and other central pathways as glutamine/glutamate metabolism preferences based on anaplerotic input to TCA or their preferential use by reductive carboxylation.

### 3.1. Breast Cancer Cell Subtypes Display a Differential Phenotype Due to Their Glycolytic Dependence

**Figure 1:**
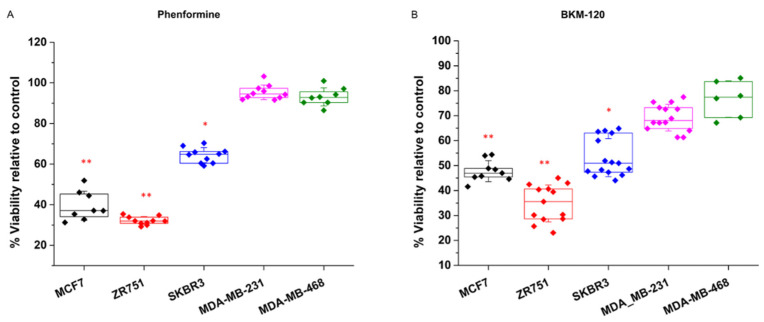
Breast cancer cell subtypes display a differential phenotype due to their glycolytic dependence. Breast cancer cell lines were treated for 96 h with metabolic drugs affecting glycolytic flux. (**A**). Cells were treated with 50 μM of the mitochondrial complex I inhibitor phenformin (Phen), which causes an imbalance in cytosolic NAD+ availability and then a reduction in glycolytic capacity. (**B**). Cells were treated with 200 nM of the PI3K inhibitor BKM-120, which consequently affects glycolytic enzymatic activity via PI3K-Akt inhibition. Both treatments showed a significant loss of viability in MCF7 and ZR751 cell lines displaying their larger glycolytic dependence. Five independent experiments were carried out (*n* = 5), each with four replicas for each experimental condition. Data displaying the percentage of cell viability relative to untreated controls (100%) are presented as the mean ± SD. * *p* < 0.01, ** *p* < 0.001.

The breast cancer cell lines showed the same pattern of response for the two experimental strategies selected for examining their glycolytic dependence (Figure 1). For the NAD+ imbalance induced by phenformin treatments, the MCF7 and ZR-751 cell lines were sensitive and showed a decrease in cell viability of 62 and 70% respectively, whereas the MDA-MD-231 and MDA-MD-468 cells displayed resistance to the phenformin effect showing no difference relative to control (Figure 1A). Similarly, the glycolytic attenuation induced by the BMK120 treatments showed the same pattern, where MCF7 and ZR751 showed decreases in cell viability of 55% and 75%, respectively, whereas MDA-MB-231 and MDA-MB-468 were more resistant to BMK120 showing just a 32% and 30% decrease of viability respectively (Figure 1B).

Collectively, these data initially indicate two differential phenotypes for glycolytic dependence between the tested breast cancer cell lines. Eventually, the SKBR3 cell line was found to show intermediate behavior between the other two phenotypes.

### 3.2. Breast Cancer Cell Subtypes Display the Same Phenotype by Their Reliance on TCA and Mitochondrial Activity

**Figure 2:**
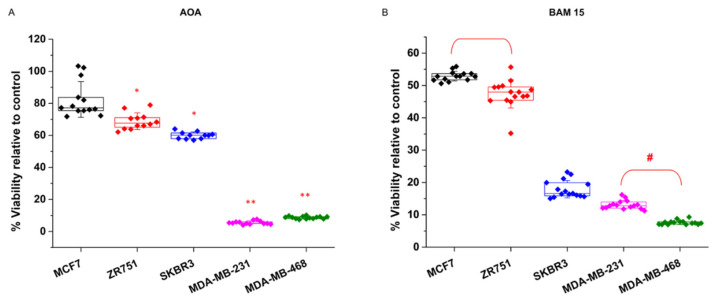
Breast cancer cell subtypes display the same phenotype by their reliance on TCA and mitochondrial activity. Breast cancer cell lines were treated for 96 h with (**A**). 1 mM aminooxyacetate (AOA), an inhibitor of transaminase activity that causes a decrease in TCA by collapsing the aspartate-malate shuttle, an imbalance in NAD+ availability and then a reduction in glycolytic capacity. (**B**) BAM15 (10 mM), a mitochondrial membrane uncoupler that produces short circuits on the mitochondrial oxidative phosphorylation capacity and ATP depletion. Both treatments showed a significant loss of viability in MDA-MB-231 and MDA-MB-468 cell lines, thus displaying their major reliance on TCA and mitochondrial oxidative activity. The HER2-positive cell line SKBR3 displayed a milder loss of viability after AOA treatment but also a stronger loss after BAM15 treatment. Five independent experiments were carried out (*n* = 5), each with four replicas for each experimental condition. Data displaying the percentage of cell viability relative to untreated controls (100%) are presented as the mean ± SD. Relative to control: * *p* < 0.01, ** *p* < 0.001, and # *p* < 0.01 relative to both MCF7 and ZR751.

Consistent with the findings described above, we further investigated whether these two phenotypes coincide with the cellular reliance on TCA and mitochondrial oxidative phosphorylation activities (Figure 2).

Treatments with the transaminase inhibitor AOA short circuits the mitochondria-cytosol transitions of reducing power conducted by the aspartate-malate shuttle. Consequently, mitochondrial TCA activity is collapsed by AOA treatment. Again, the breast cancer cell lines showed two clearly different responses to AOA (Figure 2A). The MCF7 and ZR751 cell lines were almost resistant and showed viability values of 85% and 80% relative to control, while the MDA-MB-231 and MDA-MB-468 cell lines showed a highly sensitive response, where viability filled up to 4.5% and 7% respectively (Figure 2A).

Since TCA activity inherently requires a level of mitochondrial oxidative phosphorylation over a threshold, breast cancer cell lines that present sensitivity to TCA collapse should similarly be sensitive to mitochondrial membrane uncoupling. Accordingly, breast cancer cell lines were tested for the presence of the mitochondrial membrane uncoupler BAM15. In a recently published study, we tested BAM15 collateral toxicity in control experiments and did not observe side effects on breast cancer cells. The results of the BAM15 treatments corroborated the two phenotypic differences already found for the AOA treatments (Figure 2B). The MDA-MB-231 and MDA-MB-468 cells were completely sensitive to BAM15, showing a dramatic loss of cell viability of 91% and 95% respectively, and the MCF7 and ZR751 cells displayed a milder effect. Interestingly, SKBR3 cells, which was already found to be moderately glycolytic dependent, displayed mixed behavior, with mild sensitivity to AOA (36% decrease in cell viability) (Figure 2A) and more apparent sensitivity to BAM15 (78% decrease in cell viability) (Figure 2B).

### 3.3. Breast Cancer Cells Display the Same Phenotypic Classification by Their Prevalent Glutamine/Glutamate Pathway for Nonessential Amino Acid Production

The results described above point to two potential metabophenotypes. Interestingly, the metabophenotypes correlated well with the proliferative capacity already reported for breast cancer cell lines. Moreover, the less proliferative MCF7 and ZR-751 cell subtypes displayed the same responses to the different metabolic inhibitors tested and the more proliferative MDA-MB-231 and MDA-MBD-468 cell subtypes displayed a common response between them that was clearly different from that of the other cell lines.

**Figure 3:**
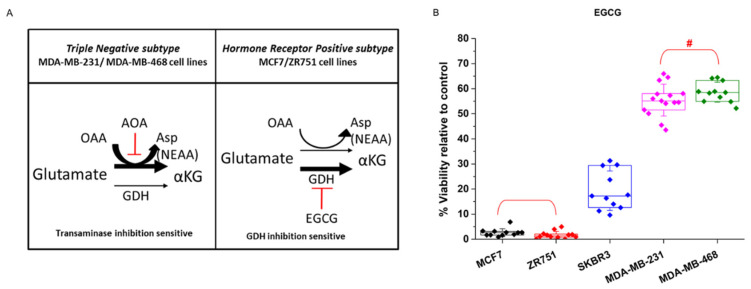
Breast cancer cells display the same phenotypic classification by their prevalent glutamate pathway supporting nonessential amino acid (NEAA) production. (**A**). Schematic representation of two different glutamate enzymatic alternatives supporting NEAA synthesis in association with the cell proliferative capacity and breast cancer subtype. The left half panel displays the preference of the highly proliferative triple-negative breast cancer cell lines MDA-MB-231 and MDA-MB-468 for transaminase versus GDH activity. The right panel displays the preference of the less proliferative hormone receptor-positive cell lines MCF7 and ZR751 for GDH versus transaminase activity. (**B**). Breast cancer cell lines were treated for 96 h with 50 μM glutamate dehydrogenase (GDH) inhibitor epigallocatechin-3-gallate (EGCG). In previous results, MCF7 and ZR751 cells displayed a clearly sensitive phenotype relative to MDA-MB-231 and MDA-MB-468 cells (resistance phenotype). The figure panel clearly shows the stronger sensitivity of MCF7 and ZR751 cells to GDH inhibition by EGCG. The HER2-positive cell line SKBR3 also displayed a significant loss of viability. Five independent experiments were carried out (*n* = 5), each with four replicas for each experimental condition. Data displaying the percentage of cell viability relative to untreated controls (100%) are presented as the mean ± SD. # *p* < 0.01 relative to both MCF7 and ZR751.

Glutamine and glutamate present several metabolic destinations and are key metabolites that sustain the production of nonessential amino acids (NEAA) that are crucial for cell proliferation. It has been recently demonstrated that glutamate is preferentially metabolized by transaminase or dehydrogenase activity depending upon the proliferative capacity of the cells (Figure 3A). Similarly, cells that present a larger proliferative capacity require a larger contribution of NEAAs, and in addition to its importance for sustaining TCA activity, transaminase activity is the preferential source of NEAAs. On the other hand, less proliferative, or even quiescent, cells that require a minor contribution of NEAA preferentially metabolize glutamate by its dehydrogenase activity (GDH) [58].

Accordingly, to test whether breast cancer cells also showed a differential response to glutamate and NEAA requirements, the cells were treated with the GDH inhibitor epigallocatechin-3-gallate (EGCG). Breast cancer cell lines displayed the expected behavior depending upon their proliferative degree, i.e., the less proliferative MCF7 and ZR751 cells were sensitive to EGCG treatment, thus displaying almost a total loss of cell viability, whereas MDA-MB-231 and MDA-MB-468 cells showed a clearly milder decrease in cell viability of 42% and 45% respectively (Figure 3B).

**Figure 4:**
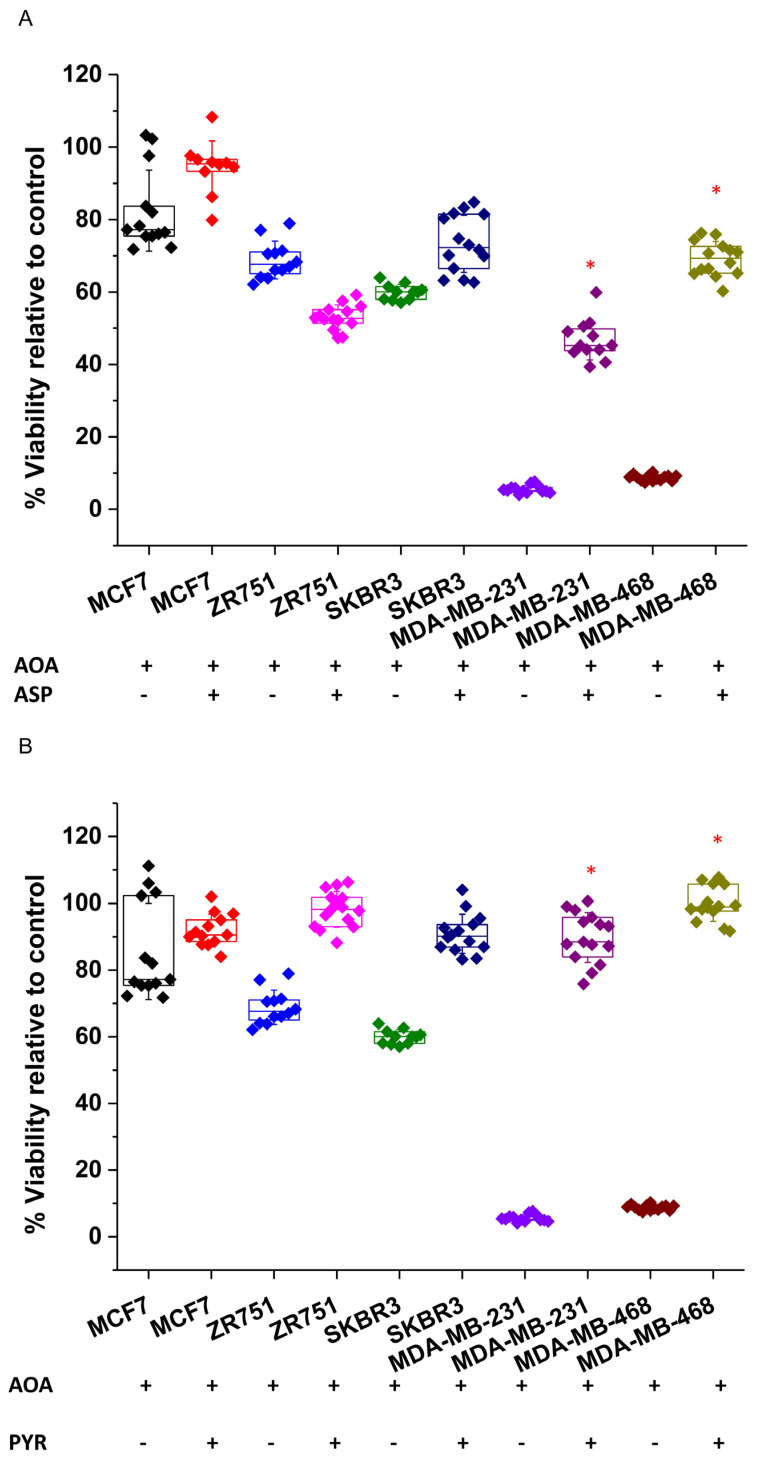
NEAA depletion by transaminase inhibition can be rescued by aspartate and pyruvate supplementation in AOA-sensitive cell lines. NEAA depletion caused by transaminase inhibition in cell lines sensitive to AOA treatments could be rescued by (**A**) 20 mM aspartate or (**B**) 2 mM pyruvate supplementation. Five independent experiments were carried out (*n* = 5), each with four replicas for each experimental condition. Data displaying the percentage of cell viability relative to untreated controls (100%) are presented as the mean ± SD. * *p* < 0.01 relative to the same cell line treated with 1mM AOA but not rescued by aspartate or pyruvate.

As already shown in Figure 2B, MDA-MB-231 and MDA-MB-468 cells were very sensitive to transaminase inhibition by AOA treatment. Importantly, corroborating their reliance on transaminase activity to sustain NEAAs, the addition of aspartate to AOA-treated MDA-MB-231 and MDA-MB-468 cells completely recovered the cell viability (Figure 4A). In addition, AOA-treated MDA-MB-231 and MDA-MB-468 cells were also successfully rescued by pyruvate, which can support aspartate synthesis [59] (Figure 4B).

Among these two metabophenotypes, the SKBR3 cell line interestingly seemed to confirm a different behavior in response to metabolic inhibition. On the one hand, it showed a milder dependence on glycolysis than the cell lines MCF7 and ZR751, as indicated by its intermediate level of sensitivity to phenformin and BMK120 treatments between the other two cellular metabophenotypes (Figure 1). On the other hand, SKBR3 behaved as MCF7 and ZR751 cells regarding transaminase inhibition resistance (Figure 2A) but GDH inhibition sensitivity (Figure 3B). However, SKBR3 showed a similar dependence on mitochondrial uncoupling, thus displaying sensitivity to BAM15 treatments as MDA-MB-231 and MDA-MB-468 cell lines (Figure 2B).

### 3.4. Breast Cancer Cell Subtypes Can Be Classified into Different Metabophenotypes in Association with the Current Clinical Classification

In summary, breast cancer cell lines showed two clearly differentiated metabophenotypes (Table 1. MCF7 and ZR751 cells showed stronger glycolytic dependence as a result of their sensitivity to phenformin and BMK120 treatments. These cell lines were resistant to transaminase inhibition but displayed high sensitivity to GDH inhibition by EGCG, which was expected for fewer proliferative cells with less demand for NEAAs. Interestingly, these cell lines are representative of the hormone receptor-positive breast cancer clinical subtypes, which are eventually associated with better prognosis and less aggressive phenotypes. On the other hand, the MDA-MB-231 and MDA-MB-468 cell lines showed major reliance on TCA and mitochondrial oxidative metabolism, as indicated by their sensitivity to the mitochondrial uncoupler BAM15 and AOA treatments. Accordingly, the cells showed a milder effect on phenformin and BMK120 treatments. In addition, these cell lines were highly sensitive to transaminase inhibition but resistant to GDH inhibition by EGCG, which was correlated with their higher proliferative capacity and thus dependent on NEAA production (Table 1). Of note, MDA-MB-231 and MDA-MB-468 cells are representative of the triple-negative breast cancer clinical subtype (negative for hormone receptor and HER2 expression), which is associated with a poor prognosis and a more aggressive phenotype. Interestingly, the SKBR3 cell line, which is representative of a different clinical subtype (negative for hormone receptors but positive for HER2 expression), showed different responses that could correlate with one of the metabophenotypes (Table 1).

### 3.5. Differential Reliance on Glycolytic or TCA Activity Observed in Breast Cancer Metabophenotypes Is Directed by Pyruvate-Predominant PC or PDH Activity

Next, further experiments were performed to investigate the metabolic mechanisms, in terms of dynamic metabolic flux, that potentially generated the metabophenotypes found in this work. Consequently, ^13^C-pyruvate isotope cell labeling experiments were performed to decipher whether the major dependence on glycolysis or TCA activity already observed was supported by a predominant metabolic route and/or enzymatic activity.

Pyruvate labeling pulses were chosen to perform metabolic flux dynamics to directly analyze the preferential mitochondrial destination of pyruvate. TCA reliance and the preferential biosynthetic pathways are determined by pyruvate metabolic destinations that are regulated by PDH and PC enzymatic activities. Hence, to primarily focus on these two pathways, ^13^C-pyruvate pulses were performed to investigate whether the breast cancer cell lines that already pointed to display different metabophenotypes might also show a preferential pyruvate destination via PDH or PC activities.

**Figure 5:**
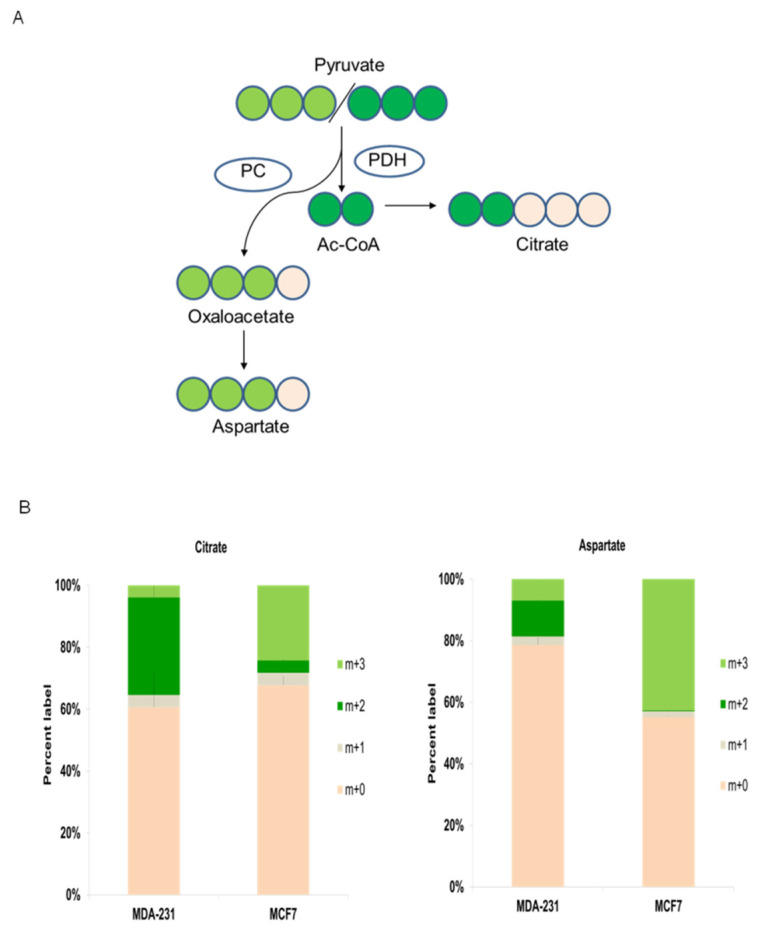
Differential reliance on glycolytic or TCA activity observed in breast cancer metabophenotypes is directed by pyruvate-predominant pyruvate carboxylase (PC) or pyruvate dehydrogenase (PDH) activity. (**A**) Schematic representation of ^13^C-pyruvate isotope cell labeling experiments with the breast cancer cell lines MCF7 and MDA-MB-231. m+3 ^13^C-pyruvate labeled with its three carbon atoms can be metabolized in mitochondria by two main pathways that are regulated by pyruvate dehydrogenase (PDH) (labeled with dark green circles) or pyruvate carboxylase (PC) (labeled with clear green circles) activities. Acetyl-CoA labeled with two carbons by PDH activity can condense the TCA with unlabeled oxalacetate (OAA) to produce m+2 citrate, which is also labeled with two carbons. On the other hand, m+3 pyruvate carboxylated by PC activity produces m+3 OAA that in turn can be transaminated to produce m+3 aspartate or condensed with unlabeled acetyl-CoA to produce m+3 citrate. (**B**) Bar graphs displaying the percentage of citrate (left panel) or aspartate (right panel) labeled species. The results clearly showed the major reliance on PDH activity and then on TCA for the triple-negative cell line MDA-MB-231 versus the stronger PC activity in the hormone receptor-positive cell line MCF7. Three independent experiments were carried out. Data display the percentage of labeled species (m+2, m+3) and unlabeled (m+0, m+1) represented as the mean ± SD.

As illustrated in Figure 5A, m+3 ^13^C-pyruvate labeled in its three carbon atoms can be metabolized in mitochondria by two main pathways that are regulated by pyruvate dehydrogenase (PDH) or pyruvate carboxylase (PC) activities. Pyruvate is decarboxylated by PDH to provide acetyl-CoA into TCA, whereas pyruvate is converted to oxalacetate (OAA) by PC, which in turn is mainly redirected to anabolic routes (Figure 5A). Acetyl-CoA labeled with two carbons by pyruvate decarboxylation can condense with unlabeled oxalacetate (OAA) to produce m+2 citrate, which is also labeled with two carbons. On the other hand, carboxylated m+3 pyruvate by PC will produce m+3 OAA (Figure 5A).

Cancer cells with a higher energetic and biosynthetic demand (via TCA anaplerotic routes) predominantly exhibit high PDH activity [60,61]. The triple-negative MDA-MD-231 8 cell line displayed active production of m+2-labeled citrate (from PDH activity) vs. m+3 citrate (from PC activity) (Figure 5B). Likewise, this cell line displayed significant amounts of m+2 aspartate generated by m+2 OAA transamination, which was previously synthesized in TCA by PDH-produced m+2 acetyl-CoA. Altogether, these results confirmed that triple-negative breast cancer MDA-B231 cells have prevalent PDH activity.

In contrast, hormone-positive MCF7 breast cancer cells, which were more dependent on glycolysis than on TCA activity, showed noticeably predominant PC activity (Figure 5A). The prevalence of PC activity was observed by the main presence of m+3 citrate and m+3 aspartate (Figure 5B), which are products of m+3 OAA produced by PC-mediated carboxylation of m+3 pyruvate, whereas a very low percentage of PDH-mediated m+2 citrate and m+2 aspartate was observed.

Importantly, these results visibly support the presence of two different metabophenotypes: the PDH predominant phenotype displaying stronger reliance on TCA activity in MDA-MB-231 triple negative breast cancer cells and the phenotype showing larger PC activity that is associated with a stronger dependence on glycolysis in MCF7 hormone receptor-positive breast cancer cells.

### 3.6. Glutamine/Glutamate Are Preferentially Metabolized by Different Pathways in Accordance with Each Breast Cancer Metabophenotype

**Figure 6:**
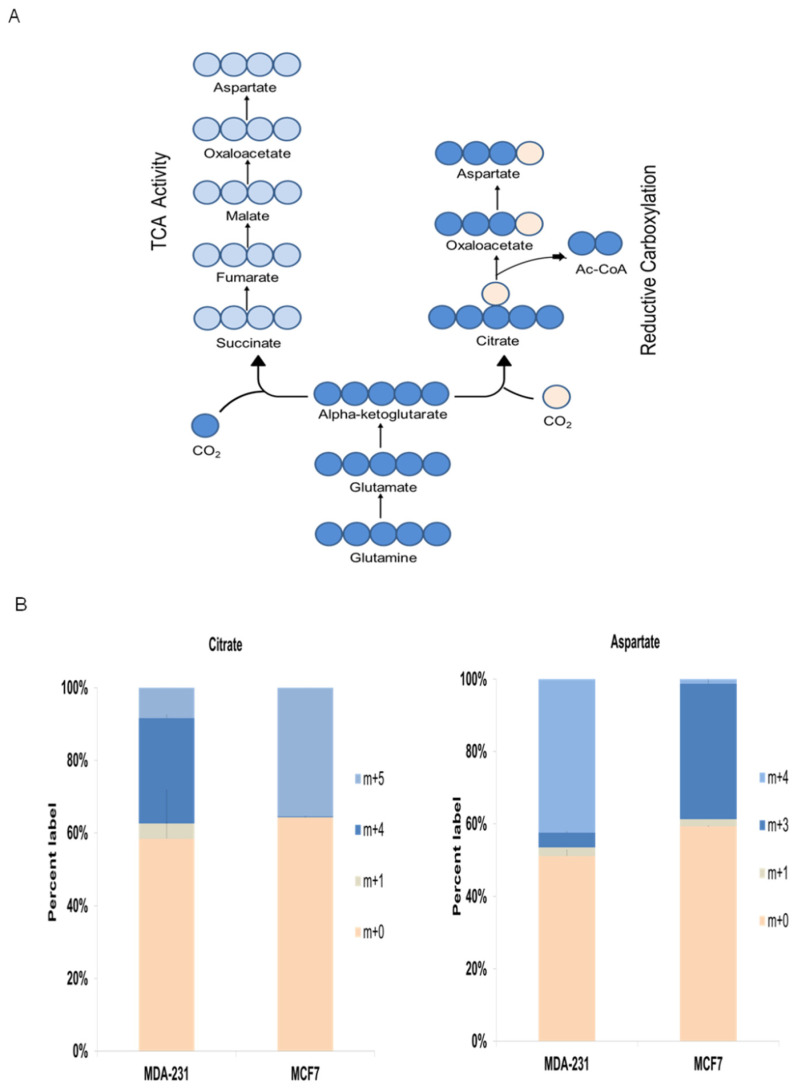
Glutamine is preferentially metabolized by different pathways in accordance with each breast cancer metabophenotype. (**A**) Schematic representation of ^13^C-glutamine isotope cell labeling experiments with the breast cancer cell lines MCF7 and MDA-MB-231. Here, m+5 glutamine is converted to m+5 glutamate, which is transformed to m+5 α-ketoglutarate. Then, mitochondrial m+5 α-ketoglutarate can enter the TCA, thus producing m+4 OAA (left branch of the scheme) that can be either condensed with unlabeled acetyl-CoA to m+4 citrate or transaminated to m+4 aspartate. On the other hand, m+5 α-ketoglutarate can be metabolized to m+5 citrate via reductive carboxylation (left branch of the scheme), which in turn will produce m+3 OAA by cytosolic citrate lyase. Finally, m+3 OAA can be transaminated to m+3 aspartate. (**B**) Bar graphs displaying the percentage of citrate (left panel) or aspartate (right panel)—labeled species. The results clearly showed the greater reliance on TCA activity and oxidative destination for glutamine in the triple-negative cell line = MDA-MB-231, as displayed by the abundant presence of m+4 citrate and m+4 aspartate. On the other hand, the hormone receptor-positive cell line MCF7 showed significant reductive carboxylation activity, as displayed by the abundant presence of m+5 citrate and m+3 aspartate. Three independent experiments were carried out. Data display the percentage of labeled (m+2, m+3) and unlabeled (m+0, m+1) species and are represented as the mean ± SD.

Finally, ^13^C-glutamine isotope cell labeling experiments were conducted to further investigate metabolic mechanisms underlying the metabophenotypes found in this work (Figure 6A). Isotope labeling data clearly showed two different preferential pathways by which ^13^C-glutamine was mainly metabolized, and these data clearly display the correlation of the two metabophenotypes with the breast cancer cell subtypes.

As illustrated in Figure 6A, m+5 glutamine is converted to m+5 glutamate, which is transformed to m+5 α-ketoglutarate. Then, mitochondrial m+5 α-ketoglutarate can enter the TCA, thus producing m+4 OAA (Figure 6A) that can be either condensed with unlabeled acetyl-CoA to m+4 citrate or transaminated to m+4 aspartate. Moreover, m+5 α-ketoglutarate can be metabolized to m+5 citrate via reductive carboxylation, which in turn will produce m+3 OAA by cytosolic citrate lyase. Finally, m+3 OAA can be transaminated to m+3 aspartate (Figure 6A).

Similar to the aforementioned data for the ^13^C-pyruvate labeling experiments, the triple-negative breast cancer cell line MDA-MB-231 displayed different ^13^C-glutamine metabolism from that shown by hormone receptor-positive MCF7 cell subtype (Figure 6B). The results supported the greater reliance on TCA activity and oxidative destination for glutamine in the triple-negative cell line = MDA-MBD-231, as displayed by the abundant presence of m+4 citrate and m+4 aspartate. On the other hand, the hormone receptor-positive cell line MCF7 showed significant reductive carboxylation activity, as displayed by the abundant presence of m+5 citrate and m+3 aspartate (Figure 6B).

Altogether, the isotope labeling results confirmed the different metabophenotypes found for triple-negative and hormone receptor-positive breast cancer cell subtypes.

## 4. Discussion

Recently, foundational contributions initiated the investigation of key metabolic features in different breast cancer cell models in vitro and in vivo. In this context, the characterization of glutamine dependence tested by differential sensitivity responses to transaminase inhibitor aminooxyacetate (AOA) was of particular relevance. Interestingly, these results were in correlation with the oncogene c-Myc expression levels between breast cancer cell lines representative of clinical subtypes [41]. In addition, the impact of AOA as a potential antimetabolic drug in breast cancer has been studied [42,43]. Likewise, opening works have shown some features of molecular impact and sensitivity responses to glutamate dehydrogenase (GDH) inhibitor epigallocatechin gallate (EGCG) [44,45], and the mitochondrial respiratory complex I inhibitor phenformin [46,47,48].

Moreover, there have been important contributions performed by unlabeled metabolomic analysis to obtain quantitative data about total metabolite contain breast cancer cell models. Some noticeable results were obtained but the data were not totally concordant displaying some discrepancies [49,50,51,52]. As indeed argued by some of the authors, unlabeled metabolome content analysis cannot address quantitative dynamic metabolic flux differences [49]. Accordingly, metabolome levels can locally and/or temporally change even in the absence of an alteration in metabolic fluxes. In addition, unlabeled metabolome meta-analysis experiments with breast cancer cell lines, including hierarchical clustering and PLSDA, revealed interesting differences in glucose uptake between breast cancer subtypes. Moreover, it was also suggested possible differences in some central metabolic pathways such as aminoacid metabolism and TCA [52]. Despite the valuable knowledge provided by these results, whether breast cancer cell subtypes might display different metabophenotypes in concordance with differences in central metabolic flux dynamics remained unresolved yet. For instance, it was shown how breast cancer cell subtypes can display a common phenotype of high glycolytic uptake and even similar dependence; however, how glucose could be preferentially diverted into one or another metabolic pathways has not been precisely addressed yet [51].

Under these premises, as already discussed by the aforementioned authors, further steps to address metabolic phenotypic differences between breast cancer cell subtypes should be performed by dynamic metabolic flux analysis for concrete pathways, starting by central metabolic routes, such as glucose preferential usage and TCA reliance. Accordingly, the present work was designed to gain insights in that direction. First, to identify different functional responses (sensitivity or resistance primarily) to alterations provoked by the selected inhibitors that might be in concordance with breast cancer subtypes. Secondly, to investigate possible metabolic flux preferences by stable-isotope dynamic labeling for key targets, such as pyruvate, based on preferential PDH or PC activities to quantitatively analyze the degree of TCA reliance, and other central pathways as glutamine/glutamate metabolism.

Thus, in the present work, the main goal was to investigate whether metabolic data obtained by this combination of functional and metabolic flux analysis approaches might complement the current clinical classification of breast cancer.

Altogether, our data clearly displayed different metabophenotypes that interestingly correlated well with the clinical classifications. According to a systemic perspective, this study selected several inhibitors as key targets. Thus, the main goal was to generate a metabolic profile that could be used to search for different phenotypes and evaluate whether the potential metabophenotypes might be associated with the current breast cancer classifications. The profiling was designed to select principal metabolic pathways that support cellular function in terms of both energy and biosynthetic demands as targets. Accordingly, the metabolic dependence on glycolytic flux and/or mitochondrial oxidative metabolism via TCA activity was analyzed based on the cellular responses to small molecules that inhibit key enzymatic targets for sustaining the activity of the proposed pathways. In particular, BKM120 and phenformin were used to impact glycolysis while AOA and BAM15 were used to short circuit TCA activity and mitochondrial oxidative metabolism. These drugs have been extensively used for metabolic profiling. BKM120 is an inhibitor of PI3K activity, which in turn produces an inhibitory effect on the PI3K-Akt axis that ultimately impacts glycolysis by decreasing its flux [56,57,62]. In addition, phenformin collapses the redox balance in terms of decreasing NAD+ availability and recycling, which is important for sustaining glycolytic activity [59,63]. On the other hand, the transaminase pan inhibitor AOA has been traditionally used to target TCA activity because it can collapse the aspartate-malate shuttle [42,64]. Finally, the mitochondrial uncoupler BAM15 was used to evaluate the degree of dependence on mitochondrial oxidative phosphorylation. Of note, in a recent work, we demonstrated the feasibility of this uncoupler for metabolic studies [39].

Specifically, the cell lines MCF7 and ZR751, which are representative of the hormone receptor-positive breast cancer cell subtype, showed stronger glycolytic dependence (Figure 1). Therefore, the biosynthetic pathways would primarily be derived from glycolytic intermediates and their corresponding anabolic routes. Consistent with this, the predominant PC activity observed in these cell lines would drive the main bulk of pyruvate to support anabolic processes via OAA and citrate redistribution with a low participation of TCA activity (Figure 5). It has been recently found that cancer cells that display similar metabolic features use prominent PC activity to sustain biosynthetic pathways, where PDH and TCA activities secondarily support anabolism [61,65,66]. In line with this metabolic phenotype, MCF7 and ZR751 showed prominent PC activity, which was clearly indicated by the significant levels of m+3 citrate and m+3 aspartate obtained from the ^13^C-pyruvate pulse experiments (Figure 5). Labeled pyruvate is mainly metabolized by PC in mitochondria to oxalacetate (OAA), which in turn can produce citrate and aspartate. Accordingly, pyruvate labeling confirmed what was initially displayed: MCF7 and ZR751 cells were sensitive to BMK120 and phenformin as “antiglycolytic” drugs (Figure 1).

On the other hand, the cell lines MDA-MB-231 and MDA-MB-468, which are representative of the triple-negative subtype, displayed a different metabophenotype featuring their main reliance on TCA activity and mitochondrial oxidative metabolism. This phenotype was clearly supported by the striking sensitivity to AOA and BAM15 treatments (Figure 2), whereas both cell lines were significantly resistant to BKM120 and phenformin (Figure 1). In addition, this metabophenotype also displayed clear differences in pyruvate metabolism, as displayed by its predominant PDH activity. Consequently, the breast cancer subtype represented by MDA-MB-231 and MDA-MB-468 cell lines would in turn support anabolic processes mainly by TCA intermediates (Figure 5). Thus, the prominent PDH and TCA activity was clearly supported by the significant presence of m+2 citrate and m+2 aspartate (both originated by a pyruvate that was previously decarboxylated by PDH) (Figure 5). As previously shown, some proliferative cancer cells, such as MDA-MB-231 and MDA-MB-468 triple-negative breast cancer subtypes, primarily rely on TCA and oxidative mitochondrial activity as biosynthetic pathways to sustain proliferation [59,60,61]. Similarly, pyruvate isotope labeling confirmed the triple-negative breast cancer cell metabophenotype that was initially indicated by their sensitivity to AOA and BAM15 treatments (Figure 2).

Notwithstanding the reliability of these findings, several interesting questions remain open. In particular, the resistance of MCF7 and ZR751 cells to AOA treatments is intriguing. However, our data tentatively indicate that these cell lines might recycle cytosolic NAD+ to sustain glycolysis, even if the aspartate-malate shuttle was collapsed by AOA transaminase inhibition. Our data indicated high PC activity, which was clearly deduced from the presence of m+3 citrate and m+3 aspartate (Figure 5). Notably, metabolism that strongly relies on PC activity requires an elevated influx of pyruvate into mitochondria, which is supported by a strong glycolytic flux. In addition, the significant production of OAA by PC-catalyzed pyruvate carboxylation could produce citrate, which then would maintain the citrate shuttle very actively, thus favoring the cytosolic production of OAA (via citrate lyase activity). Eventually, OAA reduction to cytosolic malate recycles NAD+ to maintain a high glycolytic flux. On the other hand, the resistance of the MDA-MB-231 and 468 cell lines to the mitochondrial complex I inhibitor phenformin is also intriguing because the data clearly support their reliance on TCA activity. However, because of the very active TCA, some cancer cells can maintain mitochondrial respiration that bypasses complex I by respiratory complex mutations or using specific inhibitors. This adaptation is principally based on succinate dehydrogenase-derived succinate input to mitochondrial respiration complex II together with reducing power in the form of FADH_2_ [67]. Of note, we have recently shown that phenformin treatment of MDA-MB-231 cells has no effect on cell viability and mitochondrial pH remain unaffected, thus supporting viable mitochondrial respiration and proton pumping activities [39].

The SKBR3 cell line, which is representative of the HER2+ breast cancer subtype, displayed an intriguing intermediate metabolic phenotype between the other two cell lines already discussed above. Overall, as illustrated by our results (summarized in Table 1), it might be concluded that SKBR3 does not metabolically function by a highly polarized dependence on glycolytic activity or on TCA and mitochondrial activity. Consequently, it might be said that the HER2-positive breast cancer cell line SKBR3 could present a hybrid metabophenotype between the other two, thus with a moderate dependency on both glycolytic and mitochondrial activities. In line with this suggestion, recent works have demonstrated the phenomenon of metabolic plasticity [68]. Metabolic plasticity in breast cancer samples, among others, enables cancer cells to switch their metabolic phenotypes between glycolysis and mitochondrial oxidative phosphorylation. In fact, the same authors modeled gene regulation of cancer metabolism and demonstrated that cancer cells could acquire a stable hybrid metabolic in which both glycolytic and mitochondrial activities are simultaneously available to the cells [68].

Nevertheless, further studies should be performed to gain more detailed information to clarify whether SKBR3, as a representative cell line of the HER2-positive breast cancer subtype, represents another independent metabophenotype or is just a special type within one of the already well-described metabophenotypes.

## 5. Conclusions

Confirming the scope of the proposed research, this work showed that breast cancer cell subtypes present different metabophenotypes. Importantly, these metabophenotypes are clearly correlated with the current clinical classification. These results pave the way for further studies to gain a detailed understanding of many particular metabolic routes and key targets that might be beneficial to sustain the already discovered metabophenotypes. Thus, in the future, important metabolic parameters could be crucial for potential diagnostic, prognostic and therapeutic developments.

## Figures and Tables

**Table 1:** Breast cancer cell subtypes can be classified into different metabophenotypes ^1^.

	Metabophenotype 1		Metabophenotype 2		Metabophenotype ?
	Glycolytic Flux Dependency		TCA Dependency		
	MCF7	ZR751	MDA-MB-231	MDA-MB-468	SKBR3
Phenformine	S	S	R	R	R
BKM-120	S	S	R	R	S
AOA	R	R	S	S	S
BAM15	R	R	S	S	R
EGCG	S	S	R	R	S

^1^ Based on the aforementioned metabolic profiling data, breast cancer cell subtypes could be classified into different metabophenotypes that are correlated with their clinical classification. The hormone receptor-positive cell lines MCF7 and ZR751 displayed the metabophenotype 1 phenotype, which is characterized by their greater dependence on glycolytic metabolism. The triple-negative cell lines MDA-MB-231 and MDA-MB-468 displayed the clearly different metabophenotype 2 phenotype. Interestingly, the HER2-positive cell line SKBR3 displayed a mixed response, thus pointing to a possible third independent phenotype. S (sensitive), R (resistance) in terms of the loss of cell viability response to the different metabolic drugs.

## Data Availability

The dataset from this manuscript is openly available at the University of Granada repository, DIGIBUG, at the URL http://hdl.handle.net/10481/71863.

## References

[1] DeBerardinis R.J., Lum J.J., Hatzivassiliou G., Thompson C.B. (2008). The Biology of Cancer: Metabolic Reprogramming Fuels Cell Growth and Proliferation. Cell Metab..

[2] Lunt S.Y., Vander Heiden M.G. (2011). Aerobic glycolysis: Meeting the metabolic requirements of cell proliferation. Annu. Rev. Cell Dev. Biol..

[3] Vander Heiden M.G., Cantley L.C., Thompson C.B. (2009). Understanding the Warburg effect: The metabolic requirements of cell proliferation. Science.

[4] Cairns R.A., Harris I.S., Mak T.W. (2011). Regulation of cancer cell metabolism. Nat. Rev. Cancer.

[5] Hanahan D., Weinberg R.A. (2011). Hallmarks of Cancer: The Next Generation. Cell.

[6] Pavlova N.N., Thompson C.B. (2016). The Emerging Hallmarks of Cancer Metabolism. Cell Metab..

[7] Ward P.S., Thompson C.B. (2012). Metabolic reprogramming: A cancer hallmark even warburg did not anticipate. Cancer Cell.

[8] Chaneton B., Gottlieb E. (2012). Rocking cell metabolism: Revised functions of the key glycolytic regulator PKM2 in cancer. Trends Biochem. Sci..

[9] Christofk H.R., Vander Heiden M.G., Harris M.H., Ramanathan A., Gerszten R.E., Wei R., Fleming M., Schreiber S.L., Cantley L. (2008). The M2 splice isoform of pyruvate kinase is important for cancer metabolism and tumour growth. Nature.

[10] Vander Heiden M.G., Locasale J.W., Swanson K.D., Sharfi H., Heffron G.J., Amador-Noguez D., Cantley L.C. (2010). Evidence for an alternative glycolytic pathway in rapidly proliferating cells. Science.

[11] Wong N., De Melo J., Tang D. (2013). PKM2, a Central Point of Regulation in Cancer Metabolism. Int. J. Cell Biol..

[12] DeBerardinis R.J., Mancuso A., Daikhin E., Nissim I., Yudkoff M., Wehrli S., Thompson C.B. (2007). Beyond aerobic glycolysis: Transformed cells can engage in glutamine metabolism that exceeds the requirement for protein and nucleotide synthesis. Proc. Natl. Acad. Sci. USA.

[13] Wise D.R., DeBerardinis R.J., Mancuso A., Sayed N., Zhang X.-Y., Pfeiffer H.K., Nissim I., Daikhin E., Yudkoff M., McMahon S.B. (2008). Myc regulates a transcriptional program that stimulates mitochondrial glutaminolysis and leads to glutamine addiction. Proc. Natl. Acad. Sci. USA.

[14] Zhang J., Pavlova N.N., Thompson C.B. (2017). Cancer cell metabolism: The essential role of the nonessential amino acid, glutamine. EMBO J..

[15] Anastasiou D., Poulogiannis G., Asara J.M., Boxer M.B., Jiang J.K., Shen M., Cantley L.C. (2011). Inhibition of pyruvate kinase M2 by reactive oxygen species contributes to cellular antioxidant responses. Science.

[16] Tardito S., Oudin A., Ahmed S.U., Fack F., Keunen O., Zheng L., Gottlieb E. (2015). Glutamine synthetase activity fuels nucleotide biosynthesis and supports growth of glutamine-restricted glioblastoma. Nat. Cell Biol..

[17] Yang L., Achrejal A., Yeung T.L., Mangala L.S., Jiang D., Han C., Nagrath D. (2016). Targeting Stromal Glutamine Synthetase in Tumors Disrupts Tumor Microenvironment-Regulated Cancer Cell Growth. Cell Metab..

[18] Levine A.J., Puzio-Kuter A.M. (2010). The Control of the Metabolic Switch in Cancers by Oncogenes and Tumor Suppressor Genes. Science.

[19] Dang C.V., Kim J.-W., Gao P., Yustein J. (2008). The interplay between MYC and HIF in cancer. Nat. Rev. Cancer.

[20] Düvel K., Yecies J.L., Menon S., Raman P., Lipovsky A.I., Souza A.L., Triantafellow E., Ma Q., Gorski R., Cleaver S. (2010). Activation of a Metabolic Gene Regulatory Network Downstream of mTOR Complex 1. Mol. Cell.

[21] Son J., Lyssiotis C.A., Ying H., Wang X., Hua S., Ligorio M., Kimmelman A.C. (2013). Glutamine supports pancreatic cancer growth through a KRAS-regulated metabolic pathway. Nature.

[22] Vousden K.H., Ryan K.M. (2009). p53 and metabolism. Nat. Rev. Cancer.

[23] Ying H., Kimmelman A.C., Lyssiotis C.A., Hua S., Chu G.C., Fletcher-Sananikone E., DePinho R.A. (2012). Oncogenic Kras maintains pancreatic tumors through regulation of anabolic glucose metabolism. Cell.

[24] Kulkoyluoglu-Cotul E., Arca A., Madak-Erdogan Z. (2018). Crosstalk between Estrogen Signaling and Breast Cancer Metabolism. Trends Endocrinol. Metab..

[25] Prat A., Parker J.S., Karginova O., Fan C., Livasy C., I Herschkowitz J., He X., Perou C.M. (2010). Phenotypic and molecular characterization of the claudin-low intrinsic subtype of breast cancer. Breast Cancer Res..

[26] Sorlie T., Perou C.M., Tibshirani R., Aas T., Geisler S., Johnsen H., Børresen-Dale A.L. (2001). Gene expression patterns of breast carcinomas distinguish tumor subclasses with clinical implications. Proc. Natl. Acad. Sci. USA.

[27] Cappelletti V., Iorio E., Miodini P., Silvestri M., Dugo M., Daidone M.G. (2017). Metabolic Footprints and Molecular Subtypes in Breast Cancer. Dis. Markers.

[28] Choi J., Kim E.-S., Koo J.S. (2018). Expression of Pentose Phosphate Pathway-Related Proteins in Breast Cancer. Dis. Markers.

[29] Kim S., Lee Y., Koo J.S. (2015). Differential Expression of Lipid Metabolism-Related Proteins in Different Breast Cancer Subtypes. PLoS ONE.

[30] Peng X., Chen Z., Farshidfar F., Xu X., Lorenzi P.L., Wang Y., Cheng F., Tan L., Mojumdar K., Du D. (2018). Molecular Characterization and Clinical Relevance of Metabolic Expression Subtypes in Human Cancers. Cell Rep..

[31] Bernhardt S., Bayerlová M., Vetter M., Wachter A., Mitra D., Hanf V., Lantzsch T., Uleer C., Peschel S., John J. (2017). Proteomic profiling of breast cancer metabolism identifies SHMT2 and ASCT2 as prognostic factors. Breast Cancer Res..

[32] Budczies J., Pfitzner B.M., Györffy B., Winzer K.-J., Radke C., Dietel M., Fiehn O., Denkert C. (2015). Glutamate enrichment as new diagnostic opportunity in breast cancer. Int. J. Cancer.

[33] Kim S.K., Jung W.H., Koo J.S. (2014). Differential Expression of Enzymes Associated with Serine/Glycine Metabolism in Different Breast Cancer Subtypes. PLoS ONE.

[34] Poschke I., Mao Y., Kiessling R., de Boniface J. (2013). Tumor-dependent increase of serum amino acid levels in breast cancer patients has diagnostic potential and correlates with molecular tumor subtypes. J. Transl. Med..

[35] Gross M.I., Demo S.D., Dennison J.B., Chen L., Chernov-Rogan T., Goyal B., Janes J.R., Laidig G.J., Lewis E.R., Li J. (2014). Antitumor Activity of the Glutaminase Inhibitor CB-839 in Triple-Negative Breast Cancer. Mol. Cancer Ther..

[36] Guda M.R., Asuthkar S., Labak C.M., Tsung A.J., Alexandrov I., MacKenzie M.J., Prasad D.V., Velpula K.K. (2018). Targeting PDK4 inhibits breast cancer metabolism. Am. J. Cancer Res..

[37] Harrelson J.P., Lee M.W. (2016). Expanding the view of breast cancer metabolism: Promising molecular targets and therapeutic opportunities. Pharmacol. Ther..

[38] Wu Y., Sarkissyan M., McGhee E., Lee S., Vadgama J.V. (2015). Combined inhibition of glycolysis and AMPK induces synergistic breast cancer cell killing. Breast Cancer Res. Treat..

[39] Ripoll C., Roldan M., Contreras-Montoya R., Diaz-Mochon J., Martin M., Ruedas-Rama M.J., Orte A. (2020). Mitochondrial pH Nanosensors for Metabolic Profiling of Breast Cancer Cell Lines. Int. J. Mol. Sci..

[40] Ripoll C., Herrero-Foncubierta P., Puente-Muñoz V., Gonzalez-Garcia M., Miguel D., Resa S., Paredes J., Ruedas-Rama M., Garcia-Fernandez E., Martin M. (2021). Chimeric Drug Design with a Noncharged Carrier for Mitochondrial Delivery. Pharmaceutics.

[41] Korangath P., Teo W.W., Sadik H., Han L., Mori N., Huijts C.M., Wildes F., Bharti S., Zhang Z., Santa-Maria C.A. (2015). Targeting Glutamine Metabolism in Breast Cancer with Aminooxyacetate. Clin. Cancer Res..

[42] Thornburg J.M., Nelson K.K., Clem B.F., Lane A.N., Arumugam S., Simmons A., Eaton J.W., Telang S., Chesney J. (2008). Targeting aspartate aminotransferase in breast cancer. Breast Cancer Res..

[43] Yang C.S., Stampouloglou E., Kingston N.M., Zhang L., Monti S., Varelas X. (2018). Glutamine-utilizing transaminases are a metabolic vulnerability of TAZ/YAP-activated cancer cells. EMBO Rep..

[44] Mineva N.D., Paulson K.E., Naber S.P., Yee A.S., Sonenshein G.E. (2013). Epigallocatechin-3-Gallate Inhibits Stem-Like Inflammatory Breast Cancer Cells. PLoS ONE.

[45] Puig T., Vázquez-Martín A., Relat J., Petriz J., Menendez J., Porta R., Casals G., Marrero P.F., Haro D., Brunet J. (2007). Fatty acid metabolism in breast cancer cells: Differential inhibitory effects of epigallocatechin gallate (EGCG) and C75. Breast Cancer Res. Treat..

[46] Appleyard M.V., Murray K.E., Coates P.J., Wullschleger S., Bray S.E., Kernohan N.M., Fleming S., Alessi D.R., Thompson A.M. (2012). Phenformin as prophylaxis and therapy in breast cancer xenografts. Br. J. Cancer.

[47] Guo Z., Zhao M., Howard E.W., Zhao Q., Parris A.B., Ma Z., Yang X. (2017). Phenformin inhibits growth and epithelial-mesenchymal transition of ErbB2-overexpressing breast cancer cells through targeting the IGF1R pathway. Oncotarget.

[48] Liu Z., Ren L., Liu C., Xia T., Zha X., Wang S. (2015). Phenformin Induces Cell Cycle Change, Apoptosis, and Mesenchymal-Epithelial Transition and Regulates the AMPK/mTOR/p70s6k and MAPK/ERK Pathways in Breast Cancer Cells. PLoS ONE.

[49] Dubuis S., Baenke F., Scherbichler N., Alexander L.T., Schulze A., Zamboni N. (2017). Metabotypes of breast cancer cell lines revealed by non-targeted metabolomics. Metab. Eng..

[50] Lorito N., Bacci M., Smiriglia A., Mannelli M., Parri M., Comito G., Morandi A. (2020). Glucose Metabolic Reprogramming of ER Breast Cancer in Acquired Resistance to the CDK4/6 Inhibitor Palbociclib. Cells.

[51] Ogrodzinski M.P., Bernard J., Lunt S.Y. (2017). Deciphering metabolic rewiring in breast cancer subtypes. Transl. Res..

[52] Willmann L., Schlimpert M., Halbach S., Erbes T., Stickeler E., Kammerer B. (2015). Metabolic profiling of breast cancer: Differences in central metabolism between subtypes of breast cancer cell lines. J. Chromatogr. B Anal. Technol. Biomed. Life Sci..

[53] Badve S., Dabbs D.J., Schnitt S.J., Baehner F.L., Decker T., Eusebi V., Fox S., Ichihara S., Jacquemier J., Lakhani S.R. (2010). Basal-like and triple-negative breast cancers: A critical review with an emphasis on the implications for pathologists and oncologists. Mod. Pathol..

[54] I Herschkowitz J., Simin K., Weigman V.J., Mikaelian I., Usary J., Hu Z., E Rasmussen K., Jones L.P., Assefnia S., Chandrasekharan S. (2007). Identification of conserved gene expression features between murine mammary carcinoma models and human breast tumors. Genome Biol..

[55] Wills J., Edwards-Hicks J., Finch A.J. (2017). AssayR: A Simple Mass Spectrometry Software Tool for Targeted Metabolic and Stable Isotope Tracer Analyses. Anal. Chem..

[56] Hu H., Juvekar A., Lyssiotis C., Lien E., Albeck J.G., Oh D., Varma G., Hung Y.P., Ullas S., Lauring J. (2016). Phosphoinositide 3-Kinase Regulates Glycolysis through Mobilization of Aldolase from the Actin Cytoskeleton. Cell.

[57] Riley J.K., Carayannopoulos M.O., Wyman A.H., Chi M., Moley K.H. (2006). Phosphatidylinositol 3-Kinase Activity Is Critical for Glucose Metabolism and Embryo Survival in Murine Blastocysts. J. Biol. Chem..

[58] Coloff J.L., Murphy J.P., Braun C.R., Harris I.S., Shelton L.M., Kami K., Brugge J.S. (2016). Differential Glutamate Metabolism in Proliferating and Quiescent Mammary Epithelial Cells. Cell Metab..

[59] Birsoy K., Wang T., Chen W.W., Freinkman E., Abu-Remaileh M., Sabatini D.M. (2015). An Essential Role of the Mitochondrial Electron Transport Chain in Cell Proliferation Is to Enable Aspartate Synthesis. Cell.

[60] Hosios A.M., Hecht V.C., Danai L.V., Johnson M.O., Rathmell J.C., Steinhauser M.L., Vander Heiden M.G. (2016). Amino Acids Rather than Glucose Account for the Majority of Cell Mass in Proliferating Mammalian Cells. Dev. Cell..

[61] Le A., Lane A.N., Hamaker M., Bose S., Gouw A., Barbi J., Tsukamoto T., Rojas C.J., Slusher B.S., Zhang H. (2012). Glucose-Independent Glutamine Metabolism via TCA Cycling for Proliferation and Survival in B Cells. Cell Metab..

[62] Wong K.K., Engelman J.A., Cantley L.C. (2010). Targeting the PI3K signaling pathway in cancer. Curr. Opin. Genet. Dev..

[63] Gui D.Y., Sullivan L.B., Luengo A., Hosios A.M., Bush L.N., Gitego N., Davidson S.M., Freinkman E., Thomas C.J., Vander Heiden M.G. (2016). Environment Dictates Dependence on Mitochondrial Complex I for NAD+ and Aspartate Production and Determines Cancer Cell Sensitivity to Metformin. Cell Metab..

[64] Owen O.E., Kalhan S.C., Hanson R.W. (2002). The key role of anaplerosis and cataplerosis for citric acid cycle function. J. Biol. Chem..

[65] Cheng T., Sudderth J., Yang C., Mullen A.R., Jin E.S., Mates J.M., DeBerardinis R.J. (2011). Pyruvate carboxylase is required for glutamine-independent growth of tumor cells. Proc. Natl. Acad. Sci. USA.

[66] Daye D., Wellen K.E. (2012). Metabolic reprogramming in cancer: Unraveling the role of glutamine in tumorigenesis. Semin. Cell Dev. Biol..

[67] Kitazawa S., Ebara S., Ando A., Baba Y., Satomi Y., Soga T., Hara T. (2017). Succinate dehydrogenase B-deficient cancer cells are highly sensitive to bromodomain and extra-terminal inhibitors. Oncotarget.

[68] Jia D., Lu M., Jung K.H., Park J.H., Yu L., Onuchic J.N., Levine H.B. (2019). Elucidating cancer metabolic plasticity by coupling gene regulation with metabolic pathways. Proc. Natl. Acad. Sci. USA.

